# Prevalence of iron deficiency anaemia and associated factors among preterm infants aged 3–6 months attending clinics in Dodoma, Tanzania: a facility-based cross-sectional study

**DOI:** 10.3389/fped.2026.1913886

**Published:** 2026-10-21

**Authors:** Christer Mchwampaka, Dina Mahamba, Shakilu Jumanne, Mwanaidi Amiri Msuya, Shubi Matovelo

**Affiliations:** 1 Department of Pediatrics & Child Health, School of Medicine and Dentistry, The University of Dodoma, Dodoma, Tanzania; 2 Department of Pediatrics and Child Health, Muhimbili National Hospital Mloganzila, Dar es Salaam, Tanzania; 3 Department of Biochemistry and Physiology, School of Medicine and Dentistry, The University of Dodoma, Dodoma, Tanzania

**Keywords:** associated factors, Dodoma, iron deficiency anaemia, preterm infants, Tanzania

## Abstract

**Background:**

Iron deficiency anaemia (IDA) among preterm infants is a global public health problem due to its effects on development, immunity and general growth that may be irreversible. Despite the high burden of preterm births in Tanzania the prevalence and factors associated with Iron deficiency anaemia (IDA) among preterm infants remain relatively understudied. Therefore, the study aimed to determine the prevalence of iron deficiency anaemia and associated factors among preterm infants aged 3–6 Months attending clinics in Dodoma, Tanzania.

**Methods:**

A hospital-based analytical cross-sectional study was conducted from 1st August, 2023 to 1st January, 2024, among 190 preterm infants who attended clinics in Dodoma. A structured questionnaire adopted from previous study was used to collect data from study participants. A univariable and multivariable binary logistic regression analyses were used to assess the strength of association between variables. The final results from the binary logistic regression model were presented using Adjusted Odd's Ratio (AOR), 95% Confidence Interval (CI), *p* < 0.05. The data were analyzed using the Statistical Package of Social Science IBM SPSS version 26.

**Results:**

The prevalence of IDA among preterm infants was 11.58%. Factors significantly associated with IDA included very low birth weight (<1.5 kg) (AOR = 6.91, 95% CI: 1.47–32.36, *p* = 0.014), lack of iron supplementation (AOR = 6.28, 95% CI: 1.05–37.76, *p* = 0.045), multiple pregnant (AOR = 6.85, 95% CI: 1.69–27.71, *p* = 0.007), and maternal haemoglobin before delivery (AOR = 12.00, 95% CI: 2.70–53.34, *p* = 0.001).

**Conclusion:**

IDA remains an important concern among preterm infants in Dodoma. Strengthening iron supplementation and early identification of high-risk infants, particularly those with very low birth weight, multiple gestation, or maternal anaemia, may help reduce the burden of IDA.

## Introduction

Iron deficiency (ID) and iron deficiency anaemia (IDA) are common micronutrient deficiencies, particularly among vulnerable populations such as preterm infants. They may adversely affect neurodevelopment, immunity, growth, and overall child health, with some consequences potentially being irreversible (1, 2). The United Nations has recognised reducing neonatal and infant mortality associated with complications of prematurity as an important global health priority under Sustainable Development Goal 3 (3).

The World Health Organization defines preterm birth as delivery before 37 completed weeks of gestation (4). Globally, an estimated 13.4 million babies were born preterm in 2020, corresponding to 9.9% of all live births, or approximately one in every ten babies born worldwide (5, 6). The burden of preterm birth is particularly high in low- and middle-income countries, especially in Southern Asia and sub-Saharan Africa (5, 6).

Preterm infants are 26.4 times more likely to experience iron depletion and subsequently develop IDA than term infants with normal birth weight (7). This increased vulnerability is attributed to low iron stores at birth, early onset of erythropoiesis, rapid postnatal growth, iatrogenic blood loss, and limited dietary iron sources (8). Consequently, IDA may further compromise the health of preterm infants by affecting cognitive development, impairing multiple organ systems, and increasing infant mortality rates (8).

Globally, the burden of iron deficiency and iron deficiency anaemia among preterm and low-birth-weight infants varies substantially across populations and settings. Reported estimates differ according to the population studied, age at assessment, birth characteristics, iron supplementation practices, and the criteria used to define iron deficiency and anaemia (9–12).

In Brazil, iron deficiency anaemia was reported in 26.5% of very-low-birth-weight preterm infants at one year of corrected age, while a study among late-preterm infants in Turkey reported a prevalence of 42.8% (9, 10). In Sweden, iron supplementation was associated with a lower risk of iron deficiency anaemia among marginally low-birth-weight infants (11). These findings demonstrate variability in the burden of IDA among preterm and low-birth-weight infants and highlight the potential importance of appropriate iron supplementation.

In sub-Saharan Africa, available evidence on iron deficiency and anaemia has largely focused on broader populations of children and low-birth-weight infants, with fewer studies specifically examining preterm infants, with prevalence reported at approximately 60% (13). Evidence from East Africa is similarly limited. In Kenya, a study among low-birth-weight infants attending follow-up at Kenyatta National Hospital reported an IDA prevalence of 14.5% (14). Although these findings provide regional evidence, data specifically describing IDA the prevalence and associated factors of IDA among preterm infants remain insufficient (Figure 1).

**Figure 1:**
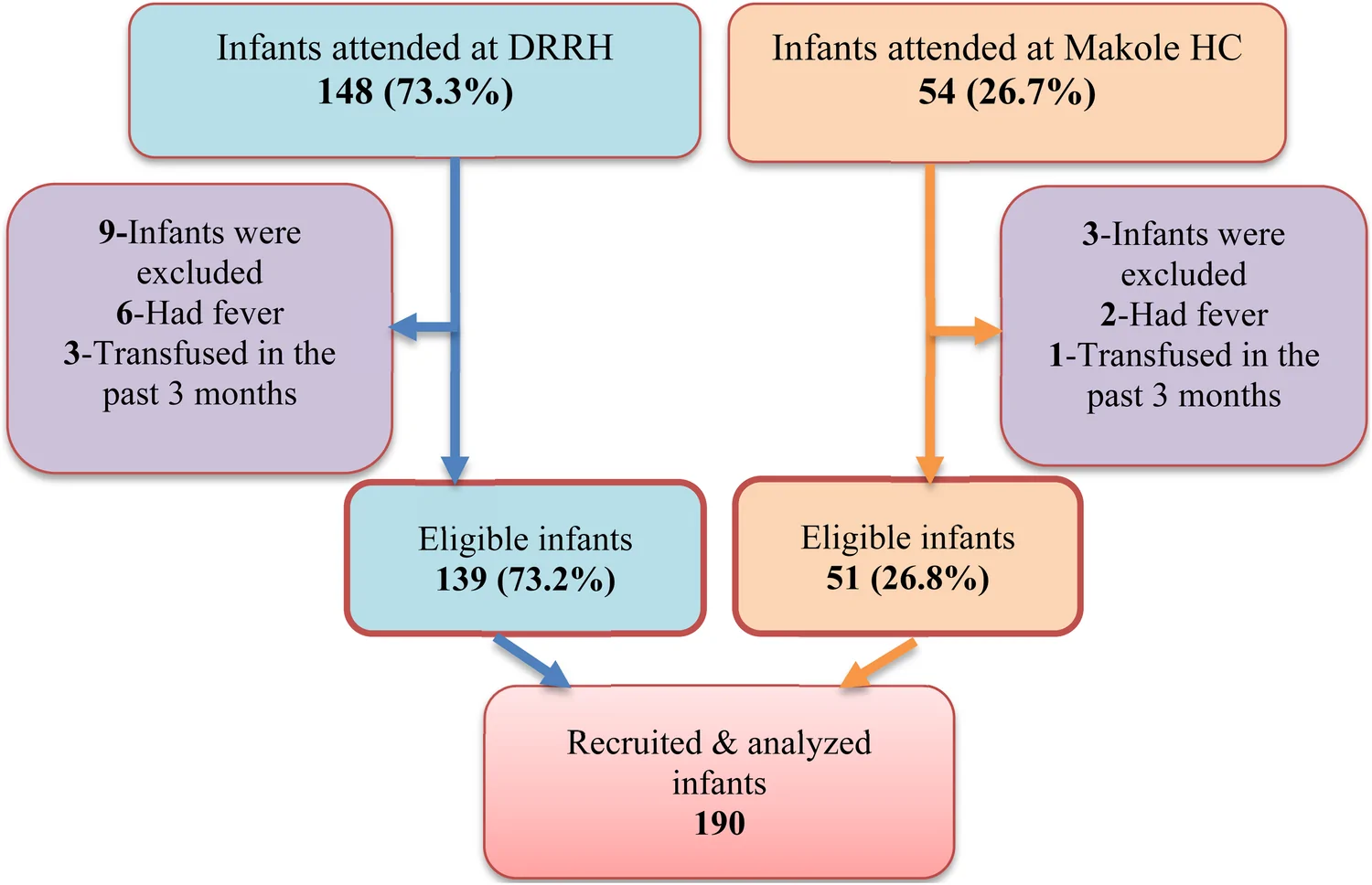
Participant flow chart showing screening, eligibility, recruitment, and inclusion in the final analysis. Source: Authors' own construction based on data collected and analysed (2024).

This evidence gap is particularly relevant to Tanzania, where preterm birth remains major public health concern. Previous studies in Tanzania have reported preterm birth prevalence rates of 14.2% and 11% (15). Preterm birth is a major contributor to neonatal and infant mortality and is associated with long-term neurodevelopmental and sensory impairments, including learning, visual, and hearing difficulties (16). However, evidence on iron deficiency anaemia (IDA) specifically among preterm infants in Tanzania remains limited, with available studies largely focusing on anaemia rather than IDA.

The development of IDA among preterm infants is influenced by several maternal and infant factors, including maternal iron deficiency, poorly controlled maternal diabetes, maternal smoking, intrauterine growth restriction, multiple gestation, fetal haemorrhage, phlebotomy-related blood loss, and inadequate or delayed iron supplementation (10). These factors are particularly important in preterm infants because substantial maternal-to-fetal iron transfer occurs during the third trimester. Preterm birth therefore interrupts this process, resulting in lower iron stores at birth, which are also closely related to birth weight (8).

Despite the established vulnerability of preterm infants to IDA, there is limited evidence on its prevalence and associated factors in Tanzania. To our knowledge, no published study has specifically assessed the prevalence of IDA and its associated factors among preterm infants attending clinics in Dodoma. This represents an important research gap because limited local evidence may hinder timely identification and management of IDA and the development of appropriate prevention strategies.

Therefore, this study aimed to determine the prevalence of iron deficiency anaemia and associated factors among preterm infants aged 3–6 months attending clinics in Dodoma, Tanzania. The findings are expected to provide locally relevant evidence to support clinical management and prevention strategies and to serve as baseline information for future research.

## Methods and materials

### Study design and setting

This facility-based analytical cross-sectional study was conducted from August 1, 2023 to January 1, 2024 at two government health facilities in Dodoma City: the paediatric clinic at Dodoma Regional Referral Hospital (DRRH) and the Reproductive and Child Health (RCH) clinic at Makole Health Centre. The facilities were selected based on their accessibility and availability of services for preterm infants. The paediatric clinic at DRRH receives approximately 88 preterm infants and provides monitoring and iron and multivitamin supplementation to infants discharged from neonatal care. The RCH clinic at Makole Health Centre receives approximately 60 preterm infants monthly and provides routine child health services, including immunization. Preterm infants requiring specialized care are referred to DRRH.

### Study population

The study population comprised all preterm infants aged 3–6 months attending clinics at DRRH and Makole health facilities during the study period.

### Inclusion and exclusion criteria

#### Inclusion criteria

All preterm infants aged 3–6 months attended the paediatric clinics for preterm infants at DRRH and the RCH clinic at Makole Health Centre.

#### Exclusion criteria

Infants were excluded if they had congenital anomalies, including cleft lip and palate, hydrocephalus, or congenital heart disease (oxygen saturation <90% with cyanosis); were acutely unwell; had tachycardia (pulse rate >160 beats/min), tachypnoea (respiratory rate >60 breaths/min), or fever (temperature ≥38 °C); or had received a blood transfusion within the previous 3 months.

### Sample size estimation

The sample size was calculated using the Kish & Leslie formula (1965) based on an estimated IDA prevalence of 14.5% reported among preterm infants with low birth weight in a previous study conducted at Kenyatta National Hospital, Kenya (14). This prevalence estimate was selected because the study assessed the same outcome in a vulnerable infant population within the East African context, where evidence on IDA among preterm infants remains limited.

$$N=\frac{Z^{2}P(1-p)}{d^{2}}$$

Where:

*N* = sample size

*Z* = score for 95% Confidence Interval which is 1.96

*P* = prevalence in a previous study

*d* = tolerable error set at 5%,

### Sampling technique

Convenience sampling was used to recruit eligible infants who presented at the selected clinics during the study period. Participants who met the predefined eligibility criteria and whose mothers or guardians signed the consent form, expressing willingness to participate in the study, were enrolled sequentially until the required sample size of 190 was reached. The same eligibility criteria and recruitment procedures were applied consistently to all participants. However, convenience sampling may introduce selection bias and limit the representativeness and generalizability of the findings beyond the selected study settings.

### Data collection tools

Data such as participants’ socio-demographic details, medical history, clinical findings, and laboratory results were collected using a structured, pre-tested questionnaire adapted from the WHO STEPS instruments (WHO STEPS, v2.1) and relevant literature, thenmodified to suit the intended study objectives. Socio-demographic data, such as place of residence, age, sex, gestational age, weight, current feeding practices, phlebotomy, and iron supplement status, were recorded. Additionally, information on maternal gestation type, the last haemoglobin level before delivery, duration of iron supplementation during pregnancy, and any maternal illnesses like pregnancy-induced hypertension, gestational diabetes, and antepartum hemorrhage were collected. Relevant details were gathered from Reproductive Child Health (RCH) cards 1 and 2. A physical examination was conducted to evaluate the infant’s health, and subsequently, a blood sample was obtained for the analysis of the parameters.

### Data sources

Data were obtained from two main sources: primary data collected directly from participants or their mothers/guardians using the study questionnaire, and relevant clinical information obtained from participants’ medical records.

### Data collection procedures

Eligible participants were identified at the selected clinics according to the predefined inclusion and exclusion criteria. After obtaining informed consent from the mothers or guardians, the researcher administered the study questionnaire and reviewed the relevant clinical records using the designated data collection forms. The same data collection procedures were followed for all participants to ensure consistency and completeness of the collected data.

### Validity and reliability

#### Validity

Validity of the questionnaire was assessed by 10 experts with relevant expertise in the study area. Each questionnaire item was rated for relevance using a five-point scale ranging from 1 (not relevant) to 5 (highly relevant), with ratings of 4 or 5 considered relevant. The Item-level Content Validity Index (CVI) was calculated as the proportion of experts who rated each item as relevant, while the Scale-level Content Validity Index based on the Average method (S-VI/Ave) was calculated as the mean of the I-CVI values across all relevant items. The tool achieved an overall S-CVI/Ave of 0.90, indicating good content validity. Items requiring improvement were revised based on expert recommendations before pre-testing.

#### Reliability

Before data collection, research assistants underwent a two-day training to standardize data collection procedures. The study instruments were pre-tested using 10% of the required sample size among participants with similar characteristics at Benjamin Hospital (BMH), Dodoma, which was outside the study area. Internal consistency of the relevant multi-items questionnaire sections was assessed using Cronbach’s alpha, with a coefficient of 0.70 or higher considered acceptable. The pre-test Cronbach’s alpha coefficient value was 0.91, indicating excellent internal consistency. The instruments were subsequently amended based on the pre-testing findings to improve clarity and consistency.

### Variable description

#### Independent variable

The independent variables in this study included infant and maternal factors. Infant-related variables were age, sex, birth weight, gestational age, number of phlebotomies since birth, current feeding practices, and iron supplementation status. Gestational age and birth weight were obtained from Reproductive and Child Health (RCH) Card 1. Maternal variables included gestation type, haemoglobin level before delivery, duration of iron supplementation during pregnancy, and maternal illnesses during pregnancy, including malaria, gestational diabetes, pregnancy-induced hypertension, and antepartum haemorrhage.

#### Dependent variable

The dependent variable was iron deficiency anaemia (IDA) among preterm infants aged 3–6 months.

#### Variable measurement and operational definition of IDA

IDA was assessed using haemoglobin (Hb), mean corpuscular volume (MCV), mean corpuscular haemoglobin concentration (MCHC), serum ferritin, and C-reactive protein (CRP). IDA was operationally defined as Hb <10 g/dL, MCV <80 fL, MCHC <32 g/dL, serum ferritin <12 µg/L, and CRP <5 mg/L. Serum ferritin <12 µg/L was used to indicate iron deficiency, consistent with WHO recommendations for children under 5 years of age (17). Serum ferritin was used to assess iron stores, while CRP was measured to account for the effect of inflammation on ferritin concentrations, as ferritin is an acute-phase reactant (18). Participants meeting at least three predefined criteria were coded as 1 (IDA), and those who did not were coded as 0 (no IDA).

### Data analysis

Data were entered into Microsoft Excel 2019, cleaned, and analysed using IBM SPSS Statistics version 26. Continuous variables were summarized using means and standard deviations (SD) or medians and interquartile ranges, as appropriate, while categorical variables were presented as frequencies and percentages. The prevalence was calculated as the proportion of enrolled preterm infants who met these criteria. Chi-square tests assessed associations between categorical variables and IDA. Variables with *p* < 0.20 in bivariate analysis were considered for the multivariable logistic regression model. Variables identified *a priori* from previous literature and those considered clinically important potential confounders were also considered for inclusion. The *p* < 0.20 threshold was used to avoid excluding potentially relevant variables at the initial screening stage. Adjusted odds ratios (AORs) with 95% confidence intervals (CIs) were recorded, with *p* < 0.05 considered statistically significant. No subgroup or interaction analyses were performed; analyses focused on the prevalence of IDA and associated factors in the overall study population.

### Ethical consideration

The study received research ethical clearance approval from the Institutional Research Review Ethics Committee (IRREC) of the University of Dodoma on 12 July 2023 with Ref. No. MA.84/261/59/154. Permission to conduct the study at Dodoma Regional Referral Hospital (DRRH) and Makole Health Centre was obtained from the respective administrations. Mothers or guardians were informed about the study objectives, procedures, potential risks, and benefits before enrolment. We obtained written informed consent before participation, and enrolled only infants whose mothers or guardians consented. For mothers or guardians unable to write, we obtained oral consent and verified it by fingerprint. Participation was voluntary, and participants could withdraw at any time without penalty or loss of routine healthcare services. Privacy and confidentiality were maintained throughout the study. Participants were identified using unique identification numbers rather than names, and no personally identifying information was included in the study dataset or reports. Study data were securely stored in password-protected files and locked storage, with access restricted to authorized members of the research team.

## Results

During the study period from August 1, 2023 to January 1, 2024, 202 preterm infants were assessed for eligibility at the two study sites. Of these, 12 were excluded due to fever (*n* = 8) or blood transfusion within the preceding 3 months (*n* = 4). A total of 190 eligible preterm infants were included in the final analysis, comprising 139 (73.2%) from Dodoma Regional Referral Hospital and 51 (26.8%) from Makole Health Centre.

### Characteristics of the study participants (*N* = 190)

Table 1 presents the characteristics of the study participants. Of the 190 preterm infants, 109 (57.37%) were female, with a mean age of 4.01 months (SD 0.99). Most infants, 157 (82.63%) were singletons, 123 (64.74%) had a birth weight of greater than or equal to 15 kg to less than or equal to 2.5 kg, and 175 (92.11%) were receiving iron supplementation. Among the maternal, 160 (84.21%) had no reported pregnancy-related illness, including pregnancy-induced hypertension (HIP), gestational diabetes, antepartum haemorrhage and malaria, while 22 (11.58%) had severe anaemia. Complete data were available for all 190 participants, with no missing data; therefore, all were included in the final analysis.

**Table 1:** Characteristics of the study participants (*N* = 190).

Variable	Label	Frequency	Percent
Infant demographic characteristics			
Age in months	3–4	131	68.95
	5–6	59	31.05
Sex	Female	109	57.37
	Male	81	42.63
Infant birth/perinatal characteristics			
Birth weight (kg)	<1.5	67	35.26
	1.5-≤2.5	123	64.74
Gestational Age (GA) at birth in weeks	<32	76	40
	32–33	40	21.05
	34–36	74	38.95
Phlebotomy since birth	None	66	34.74
	1–2 times	106	55.79
	>2 times	18	9.47
Infant clinical/management characteristics			
Current feeding practices	Exclusive breastfeeding	146	76.84
	Mixed feeding (Formula milk)	44	23.165
Iron supplementation	No	15	7.89
	Yes	175	92.11
Pallor	Pale	75	39.47
	Not pale	115	60.53
Maternal characteristics			
Gestation type	Singleton	157	82.63
	Multiple gestation	33	17.37
Duration of iron supplements in months	<3	44	23.16
	≥ 3	146	76.84
Last Hemoglobin (Hb) before delivery	<7 g/dL	22	11.58
	≥7 g/dL	168	88.42
Maternal illness during pregnancy [pregnancy-induced hypertension (PIH), gestational diabetes, antepartum hemorrhage and malaria]	Yes	30	15.79
	No	160	84.21
Laboratory findings of the infants			
Hemoglobin (Hb)	>10 g/dL	125	65.79
	<10 g/dL	65	34.21
Mean corpuscular volume (MCV) < 80fL		112	58.95
Mean corpuscular hemoglobin concentration (MCHC) < 32 g/dL		140	73.68
Serum ferritin <12 µg/L		28	14.72
C-reactive protein (CRP) < 5 mg/L		185	97.37

### Prevalence of iron deficiency anaemia among preterm infants (*N* = 190)

The prevalence of iron deficiency anaemia among preterm infants aged 3–6 months attending the selected clinics in Dodoma city was 11.58%, (Figure 2).

**Figure 2:**
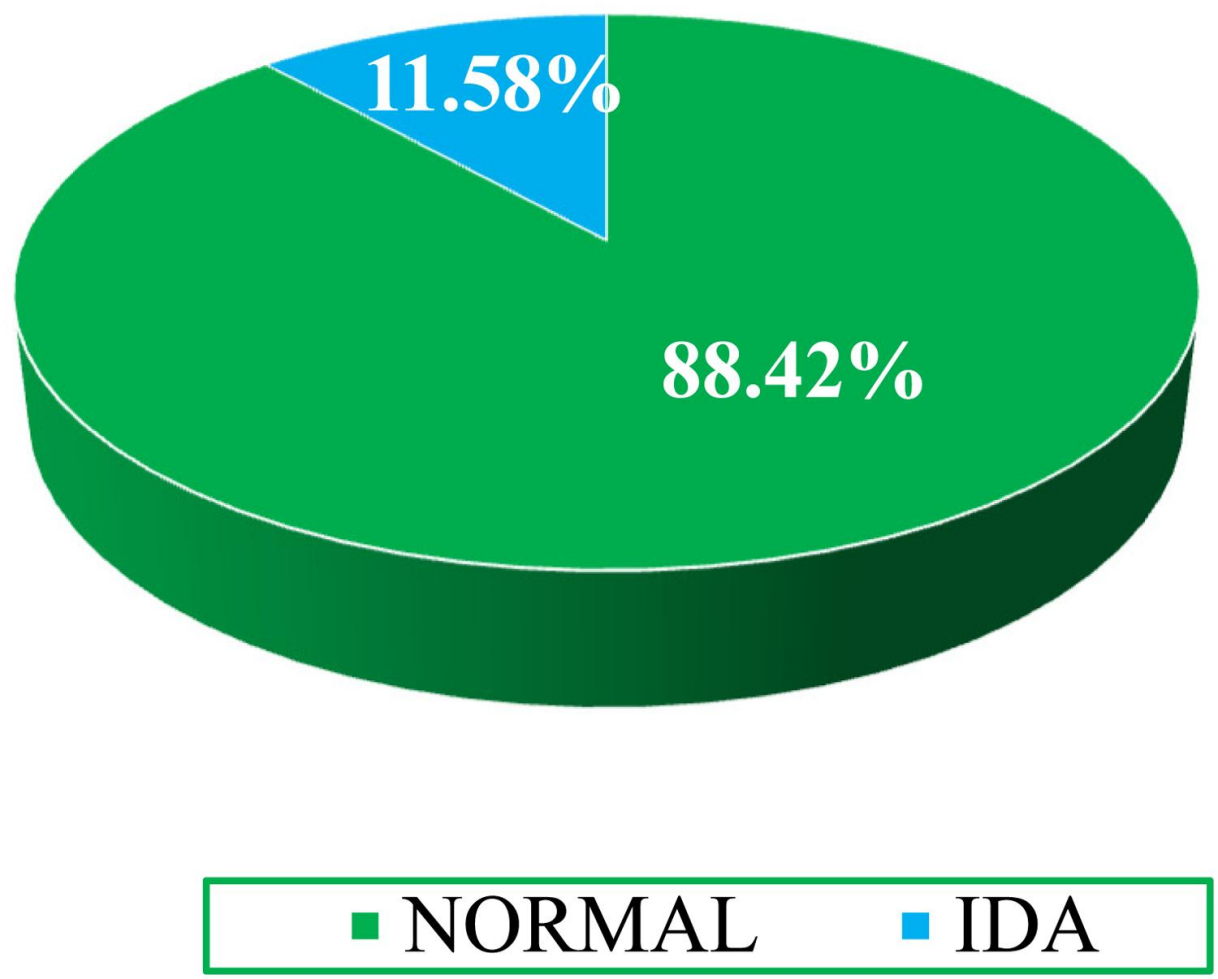
Prevalence of iron deficiency anaemia among preterm infants.

### Bivariate analysis of factors associated with iron deficiency anaemia

Bivariate analysis in Table 2 showed that birth weight, gestational age, iron supplementation, maternal gestational type, duration of maternal iron supplementation, and maternal haemoglobin level before delivery were significantly associated with IDA (*p* < 0.05). The prevalence of IDA was 19.4% among infants with very low birth weight (<1.5 kg) and 7.3% among those weighing ≥1.5 kg (*p* < 0.001). Similarly, IDA prevalence was 18.4% among very preterm infants (<32 weeks), compared with 7.5% among those born at 32–33 weeks and 6.8% among those born at 34–37 weeks (*p* = 0.017). Infants not receiving iron supplementation had an IDA prevalence of 60.0%, compared with 7.4% among those receiving supplementation (*p* < 0.001).

**Table 2:** Bivariate analysis of factors associated with iron deficiency anemia (IDA) Among preterm infants (*n* = 190).

Variables	Label	No IDA	IDA	Chi-square	*P*-Value
		n (%)	n (%)		
Infant associated factors					
Age in months	3–4	116 (88.55)	15 (11.45)	0.0068	0.9342
	5–6	52 (88.14)	7 (11.86)		
Birth weight in (kg)	<1.5	54 (80.60)	13 (19.40)	6.1881	**<0**.0001*
	1.5-≤2.5	114 (92.68)	9 (7.32)		
Gestational Age (GA) at birth in weeks	<32	62 (81.58)	14 (18.42)	5.8059	<.0.0165*
	32–33	37 (92.50)	3 (7.50)		
	34–36	69 (93.24)	5 (6.76)		
Phlebotomy since birth	None	60 (90.91)	6 (9.09)	5.1619	0.0757
	1–2 times	95 (89.62)	11 (10.38)		
	>2 times	13 (72.22)	5 (27.78)		
Feeding practices	Exclusive breastfeeding	130 (89.04)	16 (10.96)	0.2367	0.6266
	Breastfeeding with formula milk	38 (86.36)	6 (13.64)		
Iron supplementation	No	6 (40.00)	9 (60.00)	37.2950	**<0**.0001*
	Yes	162 (92.57)	13 (7.43)		
Maternal associated factors					
Gestation type	Singleton	145 (92.36))	12 (7.64)	13.6755	<.0.0010*
	Multiple gestation	23 (69.70)	10 (30.30)		
Duration of iron supplementation in Months	<3	34 (77.27)	10 (22.73)	6.9510	<.0.0084*
	≥3	134 (91.78)	12 (8.22)		
Last haemoglobin (Hb) before delivery	<7 g/dL	11 (50.00)	11 (50.00)	35.8741	**<0**.0001*
	>7 g/dL	157 (93.45)	11 (6.55)		
Maternal illness during pregnancy [pregnancy-induced hypertension (PIH), Malaria, gestational diabetes, antepartum hemorrhage]	Yes	23 (76.67)	7 (23.33)	4.8076	0.0547
	No	145(90.63)	15(9.38)		

* Statistically significant at *p* < 0.05.

*n* **-** The number of infants/participants in that category.

IDA prevalence was also higher among infants from multiple gestation with 30.3% than among those from singleton pregnant with 7.6% at *p* = 0.001. Maternal iron supplementation for less than 3 months was associated with an IDA prevalence of 22.7%, compared with 8.2% among those whose mothers received supplementation for ≥3 months (*p* = 0.008). Among infants whose mothers had severe anaemia before delivery (Hb <7 g/dL), IDA prevalence was 50.0%, while it was 6.6% among those whose mothers had Hb ≥7 g/dL (*p* < 0.001). Infant age, phlebotomy history, feeding practices, and maternal illness during pregnancy were not significantly associated with IDA (*p* > 0.05).

### Logistic regression analysis of factors associated with iron deficiency anaemia among preterm infants

Logistic regression analysis identified four factors independently associated with IDA. Very low birth weight (AOR 6.91, 95% CI: 1.47–32.36; *p* = 0.014), lack of iron supplementation (AOR 6.28, 95% CI: 1.05–37.76; *p* = 0.045), and multiple gestation (AOR 6.85, 95% CI: 1.69–27.71; *p* = 0.007) were associated with substantially increased odds of IDA. Maternal haemoglobin <7 g/dL before delivery showed the highest adjusted odds (AOR 12.00, 95% CI: 2.70–53.34; *p* = 0.001). Other factors were not independently associated with IDA after adjustment (Table 3).

**Table 3:** Logistic regression analysis of factors associated with IDA (*n* = 190).

Variable	No IDA *n* (%)	IDA *n* (%)	UNADJUSTED		ADJUSTED	
			COR [95% CI]	*P*-Value	AOR [95%CI]	*P*-Value
Birth weight(kg)						
<1.5	54 (80.60)	13 (19.40)	6.800[2.386,19.383]	0.0003	6.906[1.474,32.359]	0.014
1.5-≤2.5	114 (92.68)	9 (7.32)	Ref		Ref	
GA at birth(weeks)						
<32	62 (81.58)	14 (18.42)	3.139[1.134,8.689]	0.0276	1.988[0.450,8.784]	0.365
32–33	37 (92.50)	3 (7.50)	0.633[0.122,3.283]	0.5859	1.179[0.166,8.369]	0.869
34–37	69 (93.24)	5 (6.76)	Ref		Ref	
Phlebotomy since birth						
No	60 (90.91)	6 (9.09)	Ref		Ref	
1–2 times	95 (89.62)	11 (10.38)	1.158[0.407,3.295]	0.7835	0.369[0.082,1.664]	0.194
>2 times	13 (72.22)	5 (27.78)	3.847[1.018,14.541]	0.047	1.843[0.200,16.941]	0.589
Iron supplementations						
No	6 (40.00)	9 (60.00)	18.692[5.759,60.674]		6.282[1.045,37.763]	
Yes	162 (92.57)	13 (7.43)	Ref	<.0001	Ref	0.045
Gestation type						
Singleton	145 (92.36))	12 (7.64)	Ref		Ref	
Multiple gestation	23 (69.70)	10 (30.30)	5.255[2.037,13.554]	0.0006	6.848[1.692,27.708]	0.007
Duration of iron supplements during pregnancy (months)						
<3	34 (77.27)	10 (22.73)	3.285[1.309,8.240]	0.0113	1.835[0.452,7.457]	0.396
≥ 3	134 (91.78)	12 (8.22)	Ref		Ref	
Last Hb before delivery						
<7 g/dL	11 (50.00)	11 (50.00)	14.273[5.068,40.197]	<.0001	11.998[2.699,53.335]	0.001
≥7 g/dL	157 (93.45)	11 (6.55)	Ref		Ref	
Maternal illness during pregnancy						
Yes	23 (76.67)	7 (23.33)	2.942[1.083,7.992	0.0343	2.398[0.490,11.742]	0.281
No	145(90.63)	15(9.38)	Ref		Ref	

AOR, adjusted odds ratio; COR, crude odds ratio; CI, confidence interval; Ref, reference category. Statistical significance was set at *p* < 0.05.

## Discussion

This study found an IDA prevalence of 11.58% among preterm infants aged 3–6 months attending clinics at Dodoma Regional Referral Hospital and Makole Health Centre. Very low birth weight, lack of iron supplementation, multiple gestation, and maternal haemoglobin <7 g/dL before delivery were independently associated with IDA after adjustment for other factors.

The prevalence was comparable to the 9.9% reported in Sweden (11), and the 14.5% reported in Kenya (14). This similarity may partly reflect the vulnerability of preterm and low-birth-weight infants to iron deficiency. However, differences in study populations, age at assessment, birth weight, and iron supplementation practices should be considered when interpreting these findings.

The prevalence was lower than the 42.8% prevalence reported in Turkey (10) and the 26.5% reported in Brazil (9). These differences may partly reflect variations in gestational age, birth weight, age at assessment, and study eligibility criteria. In the present study, the high proportion of infants receiving iron supplementation may partly explain the relatively low prevalence observed. Conversely, the prevalence was higher than the 6% reported in Indonesia (4). The present study included a broader range of preterm infants, including those born before 32 weeks, whereas the Indonesian study included infants born at 32–36 weeks. Differences in gestational age and other clinical characteristics may therefore partly explain the observed variation.

### Very low birth weight and IDA

Very low birth weight (<1.5 kg) was associated with approximately seven-fold higher adjusted odds of IDA (AOR 6.91, 95% CI: 1.47–32.36). This finding is consistent with previous studies (9, 19) that reported an association between low birth weight and iron deficiency or anaemia. The association may be explained by lower iron stores at birth and increased iron requirements during rapid postnatal growth among very-low-birth-weight infants.

### Iron supplementation and IDA

Lack of iron supplementation was associated with higher odds of IDA (AOR 6.28, 95% CI: 1.05–37.76). This finding is consistent with previous evidence from (4, 20) and supports the importance of iron supplementation among preterm and low-birth-weight infants. The association is biologically plausible because preterm infants have limited iron stores and increased iron requirements during rapid growth. However, because this was a cross-sectional study, the observed association should not be interpreted as evidence of causality.

### Maternal haemoglobin and IDA

Maternal haemoglobin <7 g/dL before delivery was associated with the highest adjusted odds of IDA (AOR 12.00, 95% CI: 2.70–53.34). This finding is consistent with evidence from the Philippines and Tanzania showing an association between maternal anaemia and infant anaemia or iron deficiency (21). Maternal iron deficiency may reduce fetal iron stores, while preterm birth interrupts the substantial transfer of iron that occurs during the third trimester, potentially leaving preterm infants with lower iron stores at birth (22).

### Multiple gestation and IDA

Multiple gestation was associated with approximately seven-fold higher adjusted odds of IDA (AOR 6.85, 95% CI: 1.69–27.71). This finding is consistent with (23), who reported increased vulnerability to iron deficiency among preterm infants from multiple gestation. The association may reflect the increased maternal and fetal demand for iron during multiple gestation, together with the higher likelihood of maternal anaemia and preterm birth in these pregnancies.

### Limitations

This study has several limitations. First, convenience sampling was used to recruit eligible preterm infants, which may have introduced selection bias because infants attending the selected clinics may differ from preterm infants who did not attend these facilities. Second, the study was conducted at only two health facilities in Dodoma, which may limit the generalizability of the findings to preterm infants in other regions and healthcare settings in Tanzania. Therefore, the findings should be interpreted within the context of the study population and setting. Third, the cross-sectional design limits the ability to establish temporal or causal relationships between the identified factors and IDA. Lastly, some exposure categories contained relatively few participants, resulting in wide confidence intervals and reduced precision of some effect estimates. Nevertheless, the study used standardized data collection procedures and laboratory assessment of haemoglobin, serum ferritin, and C-reactive protein (CRP), which strengthened the assessment of IDA.

### Implications for practice and public health

These findings highlight the importance of monitoring iron status among preterm infants, particularly those with very low birth weight and those not receiving iron supplementation. Strengthening the prevention and management of maternal anaemia and ensuring appropriate iron supplementation for preterm infants may help reduce the burden of IDA in this population.

### Conclusion

This study found an 11.58% prevalence of iron deficiency anaemia (IDA) among preterm infants aged 3–6 months attending the paediatric clinic at Dodoma Regional Referral Hospital and the Reproductive and Child Health clinic at Makole Health Centre. Very low birth weight, lack of iron supplementation, multiple gestation, and maternal haemoglobin <7 g/dL before delivery were independently associated with IDA. These findings underscore the importance of appropriate iron supplementation for preterm infants and attention to maternal anaemia during pregnancy.

## Data Availability

The dataset analysed during the current study may be available from the corresponding author upon reasonable request and subject to appropriate ethical and institutional approvals.
